# Association of composite dietary antioxidant index and endometriosis risk in reproductive—age women: a cross-sectional study using big data-machine learning approach

**DOI:** 10.3389/fnut.2025.1572336

**Published:** 2025-03-27

**Authors:** Wenxin Chen, Kui Xiao, Chenyu Zhou, Jiajia Cheng, Zixuan Zeng, Fang Zhang

**Affiliations:** ^1^Department of Gynaecology and Obstetrics, Affiliated Hengyang Hospital of Hunan Normal University & Hengyang Central Hospital, Hengyang, China; ^2^Department of Plastic Surgery, Guangzhou Red Cross Hospital, Jinan University, Guangzhou, China; ^3^Reproductive Medicine Center, Hunan Provincial Maternal and Child Health Care Hospital, Changsha, China

**Keywords:** composite dietary antioxidant index (CDAI), endometriosis (EM), national health and nutrition examination survey (NHANES), cross-sectional research, machine learning

## Abstract

**Background:**

Endometriosis (EM) is a chronic gynecological disorder characterized by the growth of endometrial-like tissue outside the uterus, leading to pain and infertility. Recent studies suggest that antioxidants may play a protective role in the development of EM. However, the precise connection between the composite dietary antioxidant index (CDAI)—a key measure of dietary antioxidants—and EM risk remains unclear. This study aims to explore the relationship between CDAI and EM risk using data from the National Health and Nutrition Examination Survey (NHANES), potentially guiding dietary interventions for EM prevention.

**Methods:**

This study analyzed data from the NHANES spanning 1999 to 2006. To investigate the relationship between the CDAI and the EM, a variety of statistical techniques were employed, including a weighted multiple logistic regression model, smooth curve fitting, machine learning analysis, and subgroup analyses.

**Results:**

After controlling for potential confounding variables, the results indicated an inverse relationship between CDAI and EM (OR = 0.92, 95% CI 0.86–0.98, *p* = 0.011). Compared to participants in the lowest quartile (Q1), the odds ratios (OR) for higher CDAI in the other quartiles were as follows: Q2 (OR = 0.84, 95% CI 0.45–1.57, *p* = 0.576), Q3 (OR = 0.64, 95% CI 0.33–1.24, *p* = 0.172), and Q4 (OR = 0.47, 95% CI 0.26–0.87, *p* = 0.019). Among the various components of the CDAI, changes in vitamin A, vitamin E, and carotene were independently associated with the occurrence of EM, while both LASSO and RF machine learning algorithms consistently identified selenium and carotene as significant factors. Furthermore, subgroup analyses did not reveal significant interactions by age, body mass index, smoking, drinking, diabetes, or hypertension.

**Conclusion:**

The findings of this extensive cross-sectional study indicate a clear negative linear correlation between the CDAI and EM in American adult women. It is therefore recommended that women incorporate a greater proportion of antioxidant-rich foods into their diet to assist in the prevention of EM.

## Introduction

Endometriosis (EM), a common chronic condition where endometrial-like tissue grows outside the uterus, affects 5–10% of reproductive-age women worldwide (1–3). The disorder is often accompanied by severe symptoms such as intense pelvic pain, painful intercourse, and infertility (4, 5). Moreover, the annual direct and indirect medical costs associated with EM are staggering, with estimates reaching 78 billion USD (6). Despite its high prevalence, no curative treatments are available (7). There is growing evidence that oxidative stress plays a key role in the etiology of EM and that the severity of the disease often correlates with the levels of oxidants and antioxidants levels in the body (8, 9). Antioxidant supplementation has shown potential benefits in mitigating the development and progression of EM (7, 10). Although research has investigated the potential benefits of antioxidant supplementation, further studies are needed to understand its actual effectiveness in reducing the development and progression of EM.

Diet, as an essential source of nutrition, significantly influences human health and disease progression. In recent years, dietary approaches have emerged as a promising complementary therapy, applicable not only to malignancies but also to neurological degeneration disorders, immune system diseases, Cardiac disorders, and Metabolic dysfunctions (11). Diet plays a crucial role in managing oxidative stress in diseases. Antioxidant-rich foods, including fruits, vegetables, whole grains, and nuts, can neutralize free radicals in the body and reduce oxidative stress-induced cellular damage. Unhealthy dietary patterns often lead to oxidative stress and are associated with many metabolic disorders. However, this can be mitigated by appropriately increasing the intake of antioxidants. Therefore, it is crucial to adopt a nutritious diet to control oxidative stress and uphold the balance of cells and tissues, which is vital in averting inflammation, chronic metabolic conditions, and cancer (12, 13).

The role of dietary antioxidants has received increasing attention in recent years, and the impact of dietary antioxidant capacity on disease has become a major focus in the field of nutritional research (14). The composite dietary antioxidant index (CDAI), which includes vitamins A, C, E, carotene, zinc, and selenium, is a reliable nutritional tool for evaluating the overall antioxidant characteristics of a diet (15). Previous research has demonstrated that higher CDAI scores are associated with reduced incidences of cardiovascular diseases, depression, and diabetes complications (16–18). However, the relationship between CDAI and EM has yet to be explored.

This study examines the relationship between the CDAI and EM through a comprehensive cross-sectional analysis. The objective is to evaluate the potential of dietary antioxidants in reducing EM risk, a promising avenue with significant clinical implications given the condition’s far-reaching impact on women’s health. The study’s innovative approach has the potential to identify dietary modifications as a cost-effective prevention strategy, with implications for public health, clinical practice, and patient self-care.

## Methods

### Study population

The National Health and Nutrition Examination Survey (NHANES) is a U.S. program aimed at evaluating the health and nutritional status of Americans (19). It utilizes a rigorous stratified, multistage probability sampling approach to gather extensive data representing a wide demographic range. This particular analysis used data from 41,474 participants collected between 1999 and 2006. The specific exclusion criteria were as follows:

- Missing information for EM; Age ≥ 45 or < 20 years old (*N* = 35,917)
- Missing data for CDAI (*N* = 1,404)
- Missing data for covariates (*N* = 1959)

The final sample size was comprised of 2,195 participants. Prior to commencement of the study, approval was granted by the Ethics Review Board of the National Center for Health Statistics (NCHS). Additionally, all participants provided written informed consent.

**Figure 1:**
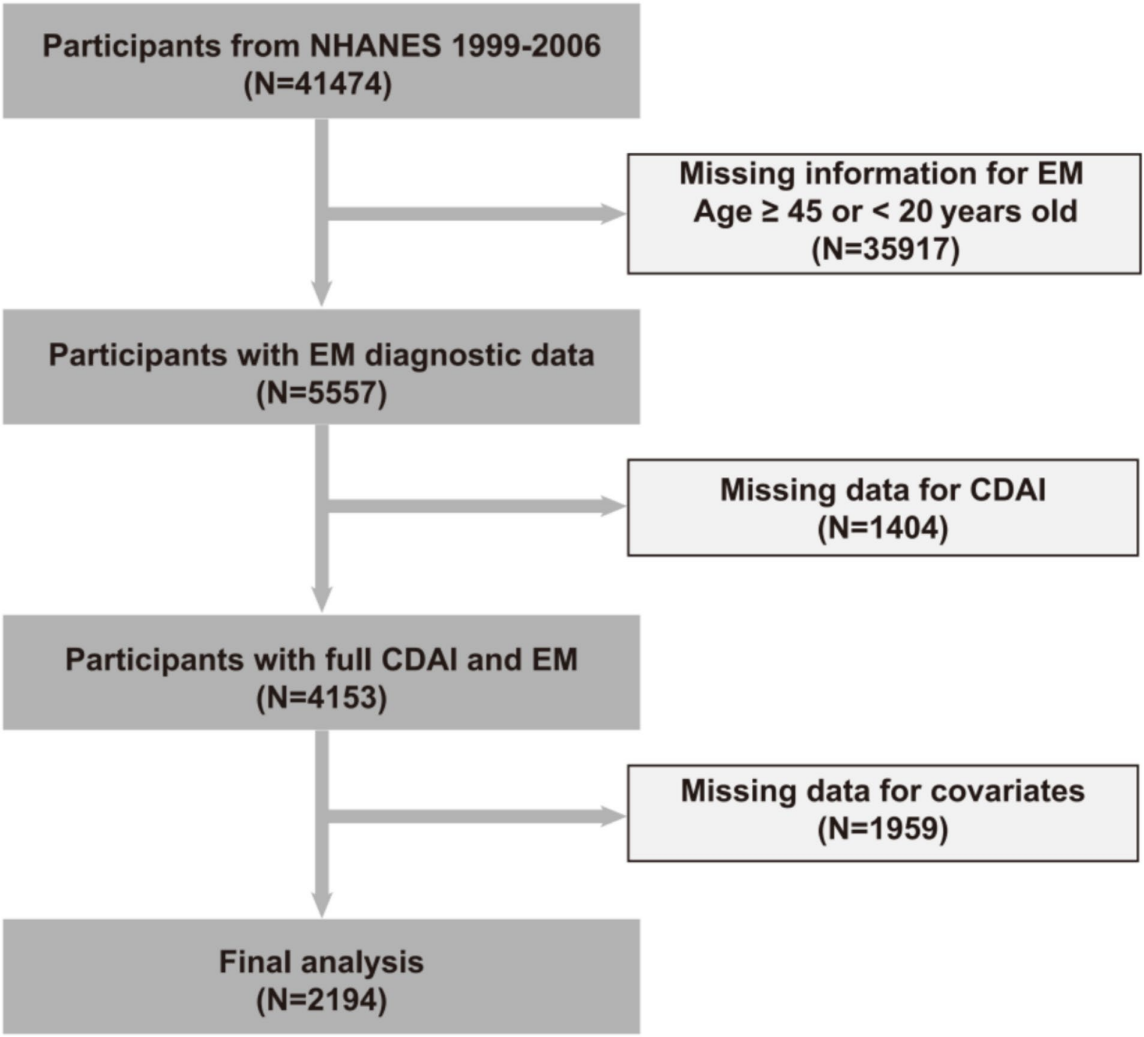
Presents a flowchart illustrating the process of participant selection in the study.

Figure 1 illustrates the full process of sample selection, depicted in a flow diagram.

### Measurement of CDAI

In the present study, CDAI was used as an exposure variable to estimate the dietary antioxidant capacity of individuals. The CDAI values were calculated based on 24-h dietary recall data from the NHANES database. The 24-h dietary recall is a valid tool widely used in epidemiologic studies to assess dietary intake and nutritional status. In the current study, all participants were given two 24-h dietary recall surveys. The first 24-h recall survey was administered face-to-face by a data collector, and the second was administered by telephone 3–10 days later. Participants were asked to recall all foods and beverages consumed the previous day. Interview data files were sent electronically from the field and imported into Survey Net, and specific nutrient intakes were calculated using the U.S. Department of Agriculture’s Food and Nutrition Database for Dietary Studies (FNDDS) (20, 21).

The CDAI was calculated based on six antioxidant components: vitamin A, vitamin C, vitamin E, zinc, selenium, and carotene. This dietary antioxidant assessment does not include antioxidants found in nutritional supplements, medications, or drinking water. To calculate the CDAI, this study followed the methodology proposed by Wright et al. (22). This study standardized the intake levels of the six antioxidants mentioned above, then subtracted the mean and divided by the standard deviation, and finally summed the standardized values of the individual nutrients to arrive at the CDAI (23). The specific formula for calculating the CDAI is as follows:


$$\mathrm{CDAI}=\overset{6}{\underset{i=1}{\sum }}\left(\frac{\mathrm{IndividualIntake}-\mathrm{Mean}}{SD}\right)$$

Additionally, a higher CDAI score indicates a greater overall dietary intake of antioxidants, reflecting a potentially more robust antioxidant defense system within the diet (24).

### Assessment of the diagnosis of EM

During four NHANES cycles from 1999 to 2006 (1999–2000, 2001–2002, 2003–2004, and 2005–2006), EM diagnoses were identified through surveys using questionnaires. Participants were asked if a physician or health professional had ever told them they had EM. Those who responded affirmatively were classified as case subjects, while those who responded negatively were placed in the control group.

### Covariates assessment

Based on prior studies and potential confounders, we identified several key covariates for our analysis, including age, education level, race, marital status (widowed/divorced/separated, married/living with partner, never married), BMI, income to poverty ratio (PIR), smoking, drinking, diabetes, and hypertension (25). Data on these variables were collected through standardized questionnaires, and participants’ weight and height were measured during physical examinations. Missing data were handled using multiple imputation techniques.

### Machine learning

To further identify the antioxidant components within CDAI that intervene in EM, this study used two machine learning algorithms: least absolute shrinkage and selection operator (LASSO), and random forest (RF).

LASSO is a regression-based method that allows the use of a large number of covariates in the model with the unique feature of penalizing the absolute value of the regression coefficients (26). In LASSO regression analysis, the Lambda value affects the choice of variables in the model. The smaller the Lambda value, the more complex the model, and the larger the Lambda value, the simpler the model. Therefore, cross-validation is needed to select the appropriate Lambda values. In this study, the R package “glmnet” is used to perform 10-fold cross-validation to avoid overfitting and underfitting problems, and to identify significant antioxidant components.

RF is an integrated learning method that improves prediction accuracy and robustness by aggregating predictions from multiple decision trees (27). Specifically, during training RF is able to randomly select features from the feature set and construct a large number of decision trees, each trained on an independent subset of samples. In a nutshell, it selects multiple samples from the sample set as the training set by back sampling and generates a decision tree from the sample set obtained from the sampling. At each generated node, features are selected randomly and without repetition. In this study, the dietary intake matrix was used as input data for the analysis of the RF Model, which utilizes 500 decision trees while analyzing the impact of each antioxidant components and screening for important antioxidant components.

### Statistical analysis

In this study, we standardized the data according to NCHS standards in the United States, categorizing participants into EM-diagnosed and non-diagnosed groups. The baseline characteristics of the population were described using mean ± standard deviation for continuous variables and frequencies and percentages for categorical variables.

The relationship between CDAI and EM was examined using logistic regression. First, the relationship between CDAI, a continuous variable, and EM was examined to ascertain whether a linear correlation exists. Secondly, to test for a *p*-value for trend, the CDAI was categorized into four groups according to quartile calculations, with the median of each group included as a continuous variable in the logistic regression. Moreover, restricted cubic spline (RCS) techniques were utilized to assess the linearity of the association between CDAI and EM. Subgroup analyses were conducted to further investigate this relationship, stratifying by variables such as age, BMI, smoking, drinking, diabetes, and hypertension.

All statistical analyses were analyzed statistically using R software (Version 3.6), and a *p*-value of less than 0.05 was considered to represent a statistically significant result.

## Results

### Characteristics of participants

A total of 2,194 participants were included in the study, with an average age of 32.64 ± 0.20 years. Of these, 6.8% had been diagnosed with EM. The baseline characteristics of the participants are presented in Table 1. A number of statistically significant (*p* < 0.05) differences were observed between the groups with respect to several variables. These included age, education level, race, marital status, PIR, CDAI, smoking, and hypertension.

**Table 1:** Characteristics of the study population based on the presence of EM.

Variable	Total	Non-EM	EM	*p*
N	2,194	2043	151	
Age, years	32.64 ± 0.20	32.26 ± 0.21	36.55 ± 0.52	< 0.001
Education level, *n* (%)				< 0.001
Below high school	143 (3.22)	141 (3.49)	2 (0.38)	
High school	828 (34.49)	763 (33.42)	65 (45.50)	
Above high school	1,223 (62.3)	1,139 (63.0)	84 (54.12)	
Race, *n* (%)				<0.0001
Mexican American	490 (9.05)	477 (9.66)	13 (2.84)	
Non-Hispanic Black	491 (12.74)	468 (13.33)	23 (6.64)	
Non-Hispanic White	1,022 (67.4)	914 (65.69)	108 (86.08)	
Other Hispanic	92 (5.08)	88 (5.39)	4 (1.96)	
Other Race	99 (5.63)	96 (5.93)	3 (2.49)	
Marriage, *n* (%)				< 0.001
Widowed/Divorced/Separated	267 (11.79)	241 (11.44)	26 (15.41)	
Married/Living with partner	1,334 (63.3)	1,231 (62.3)	103 (73.30)	
Never married	593 (24.89)	571 (26.21)	22 (11.28)	
BMI, kg/m^2^	27.61(0.2)	27.60 (0.25)	27.70 (0.52)	0.855
PIR	2.79 (0.05)	2.75 (0.05)	3.24 (0.17)	0.011
CDAI	−0.06 (0.08)	0.01 (0.08)	−0.78 (0.24)	0.003
Smoking, *n* (%)				0.010
Now	546 (27.38)	497 (26.61)	49 (35.35)	
Never	1,386 (58.8)	1,310 (59.9)	76 (47.29)	
Former	262 (13.78)	236 (13.43)	26 (17.36)	
Drinking, *n* (%)				0.391
No	673 (26.36)	635 (26.58)	38 (24.08)	
Mild	990 (47.56)	911 (47.02)	79 (53.13)	
Heavy	531 (26.08)	497 (26.40)	34 (22.79)	
Diabetes, *n* (%)				0.249
No	2099 (96.3)	1952 (96.1)	147 (98.04)	
Yes	95 (3.69)	91 (3.86)	4 (1.96)	
Hypertension, *n* (%)				0.003
No	1854 (85.5)	1743 (86.3)	111 (77.19)	
Yes	340 (14.49)	300 (13.68)	40 (22.81)	

Data are presented as mean (SD) or n (%). EM, endometriosis; PIR, Income to poverty ratio; CDAI, composite dietary antioxidant index; BMI, body mass index.

### The association between CDAI and EM

**Figure 2:**
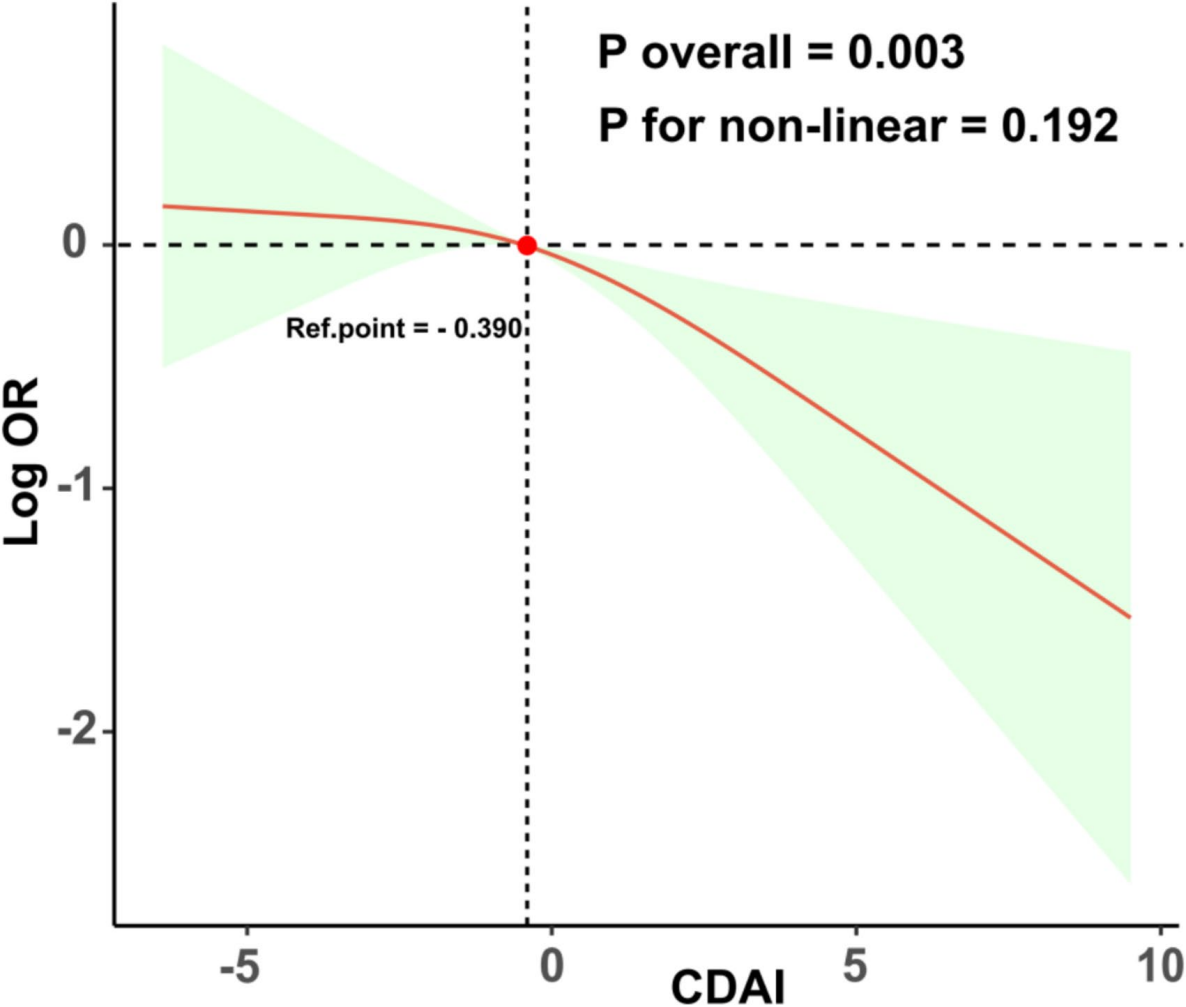
Displays the dose–response relationships between the CDAI and EM. The regression analysis utilized RCS and was adjusted for various confounding factors including age, race, PIR, education level, marital status, BMI, smoking, drinking, diabetes, and hypertension.

As illustrated in Table 2, the logistic regression weighted model revealed a correlation between the CDAI and the likelihood of EM. In Models 1, 2, and 3, there was a significant negative correlation between CDAI and the prevalence of EM (OR = 0.92; 95% CI 0.87–0.98, *p* = 0.007); (OR = 0.91; 95% CI 0.86–0.98, *p* = 0.008); (OR = 0.92; 95% CI 0.86–0.98, *p* = 0.011). These findings indicate that as CDAI increases, the prevalence of EM decreases. Following the transformation of CDAI scores into quartiles, it was observed that individuals in the highest quartile exhibited a 63% reduced likelihood of experiencing EM in comparison to those in the lowest quartile (OR = 0.47; 95% CI 0.26–0.87, *p* = 0.019). Additionally, the trend analysis yielded a statistically significant result (P for trend = 0.014). As depicted in Figure 2, the dose - response curve analysis implemented with RCS reveals a remarkable non-linear association between the CDAI and the risk of developing EM (P overall = 0.003; P for non-linear = 0.192; Ref. point = −0.390).

**Table 2:** The weighted logistic regression analysis of the association between CDAI and EM.

	Model 1		Model 2		Model 3	
	OR (95%CI)	*p*	OR (95%CI)	*p*	OR (95%CI)	*p*
CDAI	0.92 (0.87,0.98)	0.007	0.91 (0.86, 0.98)	0.008	0.92 (0.86, 0.98)	0.011
Q1	Ref		Ref		Ref	
Q2	0.85 (0.48, 1.1)	0.568	0.82 (0.45, 1.0)	0.512	0.84 (0.45, 1.57)	0.576
Q3	0.60 (0.32, 1.2)	0.105	0.62 (0.32, 1.9)	0.146	0.64 (0.33, 1.24)	0.172
Q4	0.48 (0.27, 0.6)	0.014	0.46 (0.26, 0.3)	0.011	0.47 (0.26, 0.87)	0.019
P for trend		0.009		0.009		0.014

Model 1: no adjusted. Model 2: adjusted for age, education level, race, marital status, and PIR. Model 3: adjusted for age, education level, race, marital status, BMI, PIR, smoking, drinking, diabetes, and hypertension. CDAI, composite dietary antioxidant index; EM, endometriosis; PIR, poverty income ratio; BMI, body mass index. CDAI: Q1 (<−2.426), Q2 (−2.426, −0.462), Q3 (−0.462, 1.868), and Q4 (≥1.868).

### The association between CDAI components and EM by weighted logistic regression

A further analysis was conducted to investigate the association between the six antioxidant components of CDAI and EM. As shown in Table 3, following the adjustment for all variables, only vitamin A (OR = 0.999, 95% CI 0.999–1.000, *p* = 0.17), vitamin E (OR = 0.934, 95% CI 0.885–0.986, *p* = 0.015), and carotene (OR = 1, 95% CI 1.000–1.000, *p* = 0.034) were independently associated with the presence of EM.

**Table 3:** The weighted logistic regression analysis of the association between CDAI components and EM.

	Model 1		Model 2		Model 3	
	OR (95%CI)	*p*	OR (95%CI)	*p*	OR (95%CI)	*p*
Vitamin A (μg)	0.999 (0.999, 1.00)	0.038	0.999 (0.999, 1.000)	0.014	0.999 (0.999, 1.000)	0.017
Vitamin C (mg)	0.997 (0.994, 0.99)	0.015	0.998 (0.996, 1.001)	0.130	0.998 (0.996, 1.001)	0.171
Vitamin E (mg)	0.949 (0.902, 0.99)	0.044	0.935 (0.887, 0.986)	0.015	0.934 (0.885, 0.986)	0.015
Zinc (mg)	0.991 (0.957, 1.06)	0.601	0.989 (0.952, 1.027)	0.559	0.989 (0.950, 1.030)	0.595
Selenium (μg)	0.997 (0.992, 1.03)	0.346	0.998 (0.992, 1.004)	0.542	0.998 (0.992, 1.005)	0.594
Carotene (μg)	1.000 (1.000, 1.00)	0.033	1.000 (1.000, 1.000)	0.036	1.000 (1.000, 1.000)	0.034

Model 1: no adjusted. Model 2: adjusted for age, education level, race, marital status, and PIR. Model 3: adjusted for age, education level, race, marital status, BMI, PIR, smoking, drinking, diabetes, and hypertension. CDAI, composite dietary antioxidant index; EM, endometriosis; PIR, poverty income ratio; BMI, body mass index.

### Identification of key CDAI components associated with EM by machine learning

**Figures 3:**
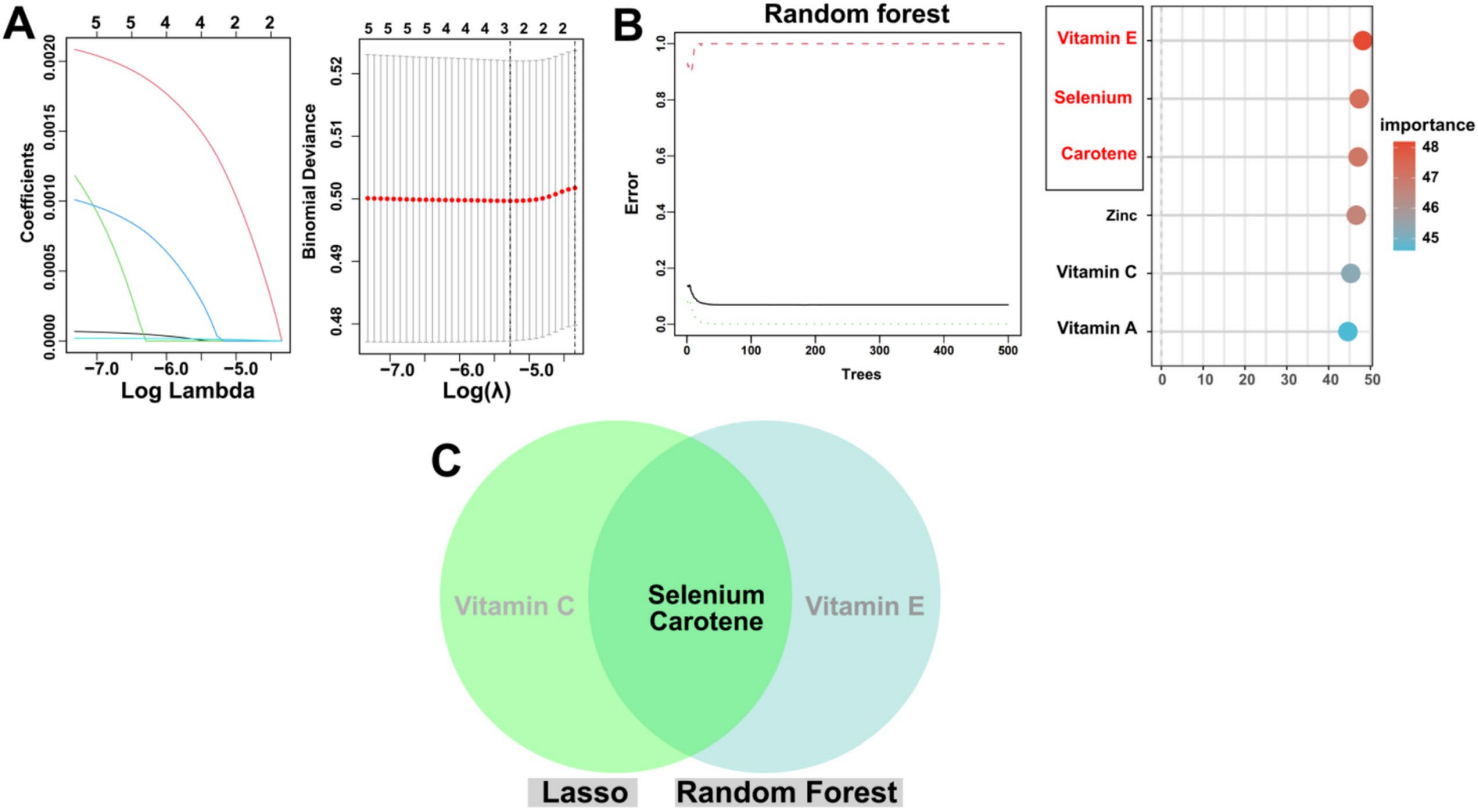
Illustrates the identification of key CDAI components associated with EM using LASSO and RF methodologies. **(A)** The left panel displays the LASSO coefficient profiles of the 6 CDAI components, while the right panel shows the results after cross-validation for tuning parameter selection, where 3 CDAI components (vitamin C, selenium, and carotene) were identified as significant antioxidant components. **(B)** The RF algorithm identified 3 CDAI components (vitamin E, selenium, and carotene), as depicted in the left panel showing the RF error rate versus the number of classification trees, and the right panel showcasing the importance scores of these components. **(C)** The Venn diagram demonstrates the overlap of key CDAI components identified by both the LASSO and RF algorithms.

In the present study, we conducted a more detailed investigation into the relationship between the six antioxidant components of CDAI and EM, employing two distinct machine learning algorithms. Figure 3A depicts the outcomes of the LASSO algorithm, which identified vitamin C, selenium, and carotene as significant antioxidant components. Figure 3B illustrates the results of the RF algorithm, which similarly highlighted vitamin E, selenium, and carotene as significant antioxidant components. Notably, both the LASSO and RF algorithms identified selenium and carotene as significant antioxidant components, as illustrated in Figure 3C.

### Subgroup analysis

**Figure 4:**
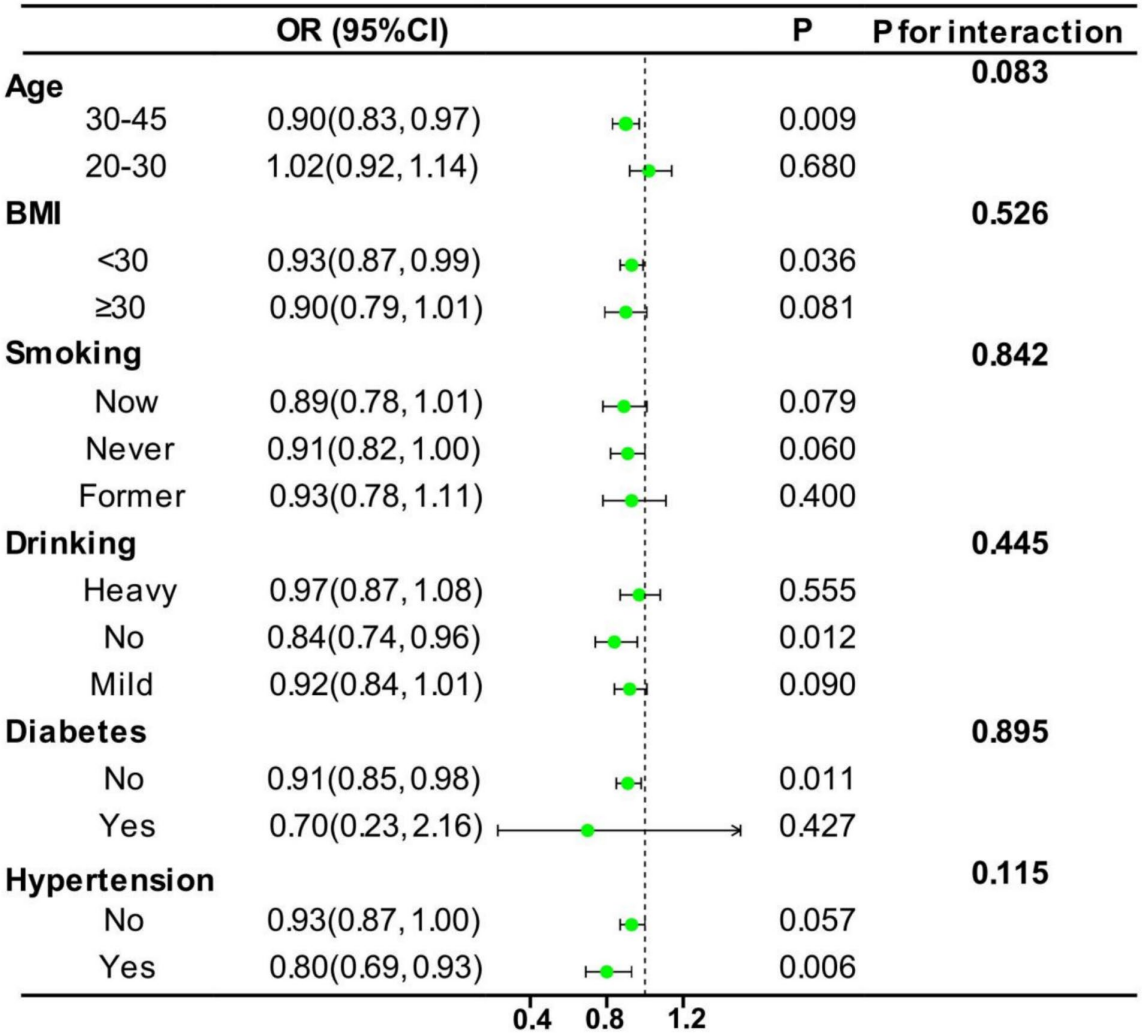
Subgroup analysis for the association between CDAI and EM. The analyses were stratified by age (20–30 and 30–45 years), BMI (<30 kg/m^2^ and ≥ 30 kg/m^2^), smoking status (new, never, and former), drinking status (heavy, none, and mild), diabetes (yes and no), and hypertension (yes and no). Model 3, which was used in subgroup analysis, was adjusted for age, race, PIR, education level, marriage, BMI, smoking, drinking, diabetes, and hypertension.

Figure 4 presents the results of our investigation into the stability of the relationship between the CDAI and EM. To examine this relationship, the data were categorized based on age, BMI, smoking, drinking, diabetes, and hypertension. After conducting subgroup analyses, no significant interactions were observed between the CDAI and these categorized variables (all p for interaction >0.05), indicating that the observed relationship remained consistently stable. Specifically, the subgroup analysis revealed that women between the ages of 30 and 45, those with a BMI below 30, women without a history of alcohol consumption, and women with hypertension exhibited a more pronounced tendency to demonstrate benefits in response to a high CDAI. This research result is likely to indicate that, in terms of preventing EM, an antioxidant diet may be able to play a more effective preventive effect for specific populations, especially middle-aged women, those with a moderate body shape, non-drinkers, and women with hypertension.

## Discussion

A notable inverse correlation was identified between CDAI and EM risk within the US population, indicating that elevated CDAI may confer protection against EM. The findings from smooth spline fitting confirmed that this correlation was linear and consistent across demographic characteristics, suggesting a broad protective effect of CDAI. These results underscore the potential role of antioxidant-rich diets in the prevention of EM and support further investigation into the efficacy of dietary interventions for risk reduction.

Endometriosis (EM), characterized by chronic pelvic pain and infertility (28), is not only a significant cause of infertility in women but also severely affects their physical and mental health. Given its substantial impact on women’s health, it is now considered a public health issue worth further investigation (28). Current research investigators believe that EM may lead to a chronic inflammatory response and is considered one of the common gynecological diseases (29). With the continuous in-depth development of medical research, recent studies have highlighted that oxidative stress plays a crucial role in the pathogenesis and progression of EM (30). In recent years, the understanding of EM has deepened from being a common disease to becoming a worldwide health problem. The concept of treatment has shifted from “radical surgery” to focusing on “relief of pain, improvement of fertility, comprehensive treatment, and long-term management (31). Dietary treatment and prevention have emerged as one of the most crucial methods for preventing and treating EM (32).

A number of studies show that vitamin A is of great significance in the onset and progression of EM (33, 34). Lowered vitamin A levels affect endometrial cell proliferation and immune balance, promoting endometriosis (33). Moreover, *In vitro* experiments have shown that retinoic acid, a metabolite of vitamin A, can downregulate the expression level of interleukin-6 (IL-6), inhibit the epithelial-mesenchymal transition (EMT) process, and thus reduce the invasive ability of endometriosis cells (35). The above research results indicate that vitamin A and its metabolite, retinoic acid, may have a significant correlation with the regulation of immune responses and the EMT process in terms of inhibiting the occurrence of endometriosis. In addition, Nalini et al. pointed out that vitamins C can effectively reduce the levels of a variety of inflammatory markers closely related to endometriosis (35). These markers include IL-6, secreted factor (RANTES), and monocyte chemoattractant protein-1 (MCP-1). Furthermore, in *in vitro* experiments, Ozlem et al. and Hayedeh et al. further demonstrated that vitamin C has the effects of preventing the formation of and promoting the regression of endometriotic implants in a rat model of EM (36, 37). In addition, another study shows that vitamins C, E, and *β*-carotene can significantly increase the total antioxidant capacity (TAC) of endometriosis patients’ plasma. At the same time, they can reduce the level of the oxidative stress marker 8-hydroxydeoxyguanosine (8-OHdG) and alleviate DNA damage (38). In conclusion, vitamins C can reduce the levels of inflammatory markers related to the tissue of EM. On the other hand, vitamins C, E, and β-carotene can enhance the total antioxidant capacity of the plasma of patients with endometriosis, which indicates that they have potential beneficial effects in the treatment of endometriosis. Regarding the relationship between zinc and endometriosis, some studies indicates that dietary zinc intake is positively correlated with the prevalence of EM (38, 39). In addition, a study by Mier-Cabrera et al. emphasizes the role of antioxidants, including zinc, in ameliorating oxidative stress markers in women with EM. *In vitro* experiments have demonstrated that zinc can inhibit the activity of matrix metalloproteinase-9 (MMP-9) and decrease the migratory capacity of ectopic endometrial stromal cells (40). This finding not only reflects the importance of zinc in improving the endometrial internal environment but also reveals its key role in directly acting on the migratory process of ectopic cells. Notably, Oxidative stress has a dual harm in the development process of endometriosis. It can not only promote the occurrence of the inflammatory response but also further aggravate the pathological process by damaging cellular DNA and proteins (41, 42). Studies have shown that the intake of antioxidants can protect against NF-κB activation, thereby alleviating the related symptoms caused by EM (36). This indicates that oxidative stress forms a vicious cycle in the development of EM, and the intake of antioxidants is an important means to break this cycle and relieve symptoms.

The use of diet to manage EM has been a widely discussed topic in research (32, 43, 44). Interestingly, studies have found that patients with EM often have lower intakes of vitamins A, C, E, zinc, and copper (38), suggesting a link between an antioxidant-rich diet and the disease, which aligns with our own findings. Clinical trials have also noted that antioxidants (vitamins E and C) can effectively improve dysmenorrhea and pelvic pain (45–47). Antioxidant-rich diet reduces abnormal endometrial tissue growth and inflammatory response by reducing oxidative stress (48, 49). Increased ROS in patients with EM, along with trends that worsen with disease severity, have been documented (41, 42). The specific mechanisms may involve the production of inflammatory factors and mitochondrial dysfunction (30). At the same time, representative antioxidant medications such as curcumin, astaxanthin, resveratrol, and quercetin have demonstrated their potential value in the treatment of EM (50–52). Targeted interventions against oxidative stress are considered a promising strategy for inhibiting disease progression and alleviating associated chronic pain and infertility symptoms (53–55). In conclusion, the reduction of oxidative stress within the body through dietary modifications represents a crucial strategy in the fight against EM.

Previous clinical studies have mainly focused on the impact of individual nutrients on EM (25). However, the CDAI is a reliable scoring system used to quantify the antioxidant capacity of daily diets. By assessing the natural combination of nutrients in foods, CDAI can more deeply explore the synergistic effects of various antioxidants in daily diets, helping to neutralize free radicals in the body, reduce oxidative stress on cells, and potentially slow the progression of EM. Compared to single nutrient research methods, CDAI provides a more systematic and comprehensive assessment, reflecting the complexity and diversity of dietary patterns. Using CDAI also aids in developing personalized dietary intervention strategies to improve patients’ quality of life and disease management outcomes. As the understanding of the pathological mechanisms of EM continues to deepen, the importance of dietary treatment and prevention is becoming increasingly evident. Future research should further validate the clinical applicability of CDAI and explore its suitability in different female populations, providing more robust scientific evidence for the prevention and treatment of EM.

Although no significant interaction was detected in this subgroup analysis, the obtained results still have important guiding significance for clinical practice. The impact of an antioxidant diet may be more prominent within a specific population. This finding emphasizes the importance of fully considering individual differences of patients (such as age, drinking history, and hypertension history) in the clinical practice. At the same time, it also points out the direction for subsequent research, that is, to further explore and optimize the dietary strategies for specific patient groups, so as to provide a more solid theoretical support and practical guidance for clinical applications.

This study, based on NHANES data, is the first to reveal a linear relationship between CDAI and EM, while eliminating the potential for bias from other confounding factors. Nevertheless, it is important to acknowledge the existence of several limitations that require further investigation in future research. (1) Due to the limitations of the cross-sectional design, we are unable to infer causal relationships between exposure and outcome. Therefore, further validation through larger-scale prospective cohort studies is needed to confirm these findings. (2) Due to the limited sample size and relevant sample information, it is unrealistic to comprehensively explore all possible confounding factors, such as menstrual history, family history of endometriosis, and history of pelvic surgery. These unstudied factors may have led to biases in the results. (3) Most of the data regarding the diagnosis of EM were obtained from the participants of the NHANES through interviews or self-reported questionnaires. However, it should be noted that self - reporting may lead to misclassification. Some women may present symptoms similar to those of endometriosis but actually do not have the disease, while some women with the disease may not be aware of their condition or be unable to accurately report their situation. Such misclassification may distort the true relationship between CDAI and EM. To control this recall bias as much as possible, based on the above considerations, we selected women in the core age range of 20–45 years old, where the disease is most prevalent, as the research subjects. In this female group, a series of typical symptoms are more likely to present (56, 57). However, it should be noted that our study was based on self-reported data and 24-h dietary recall, so there is a possibility of recall bias. (4) It should be noted that the questionnaire used to diagnose EM did not include some participants who were under the age of 20 or over the age of 45. Consequently, the results may be more applicable to individuals aged 20–45 and not easily generalizable to those under 20 or over 45. (5) It is possible that the NHANES study population is not entirely representative of the general population.

## Conclusion

This cross-sectional analysis revealed a significant negative correlation between CDAI and the risk of EM. The finding suggests that a higher CDAI score is associated with a lower risk of EM, indicating that a diet rich in antioxidants may be related to a reduced risk of EM. Therefore, we recommend increasing the intake of foods that can improve the CDAI score, such as nuts, leafy greens, and brightly colored fruits. Meanwhile, maintaining a balanced diet that includes whole grains, lean proteins, and healthy fats is crucial for achieving overall health benefits. However, longitudinal studies are needed in the future to further clarify the causal relationship and evaluate the long-term effects of dietary interventions, providing a basis for formulating personalized dietary strategies to better support the health management of EM patients and high-risk groups.

## Data Availability

The original contributions presented in the study are included in the article/supplementary material, further inquiries can be directed to the corresponding author.

## References

[1] Taylor HS Kotlyar AM Flores VA. Endometriosis is a chronic systemic disease: clinical challenges and novel innovations. Lancet (London, England). (2021) 397:839–52. doi: 10.1016/S0140-6736(21)00389-5, PMID: 33640070

[2] Chapron C Marcellin L Borghese B Santulli P. Rethinking mechanisms, diagnosis and management of endometriosis. Nat Rev Endocrinol. (2019) 15:666–82. doi: 10.1038/s41574-019-0245-z, PMID: 31488888

[3] Giudice LC Kao LC. Endometriosis. Lancet (London, England). (2004) 364:1789–99. doi: 10.1016/S0140-6736(04)17403-5, PMID: 15541453

[4] Allaire C Bedaiwy MA. Yong PJ: diagnosis and management of endometriosis. CMAJ: Canadian Med Assoc J = journal de l'Association medicale canadienne. (2023) 195:E363–e371. doi: 10.1503/cmaj.220637, PMID: 36918177 PMC10120420

[5] de Ziegler D Borghese B Chapron C. Endometriosis and infertility: pathophysiology and management. Lancet (London, England). (2010) 376:730–8. doi: 10.1016/S0140-6736(10)60490-4, PMID: 20801404

[6] Evans S Fernandez S Olive L Payne LA Mikocka-Walus A. Psychological and mind-body interventions for endometriosis: a systematic review. J Psychosom Res. (2019) 124:109756. doi: 10.1016/j.jpsychores.2019.109756, PMID: 31443810

[7] Zheng SH Chen XX Chen Y Wu ZC Chen XQ Li XL. Antioxidant vitamins supplementation reduce endometriosis related pelvic pain in humans: a systematic review and meta-analysis. Reproductive Biolog Endocrinol: RB&E. (2023) 21:79. doi: 10.1186/s12958-023-01126-1, PMID: 37644533 PMC10464024

[8] Cacciottola L Donnez J Dolmans MM. Can endometriosis-related oxidative stress pave the way for new treatment targets? Int J Mol Sci. (2021) 22:7138. doi: 10.3390/ijms22137138, PMID: 34281188 PMC8267660

[9] Clower L Fleshman T Geldenhuys WJ Santanam N. Targeting oxidative stress involved in endometriosis and its pain. Biomol Ther. (2022) 12:1055. doi: 10.3390/biom12081055, PMID: 36008949 PMC9405905

[10] Markowska A Antoszczak M Markowska J Huczyński A. The role of selected dietary factors in the development and course of endometriosis. Nutrients. (2023) 15:2773. doi: 10.3390/nu15122773, PMID: 37375677 PMC10303755

[11] Xiao YL Gong Y Qi YJ Shao ZM Jiang YZ. Effects of dietary intervention on human diseases: molecular mechanisms and therapeutic potential. Signal Transduct Target Ther. (2024) 9:59. doi: 10.1038/s41392-024-01771-x, PMID: 38462638 PMC10925609

[12] Jiang S Liu H Li C. Dietary regulation of oxidative stress in chronic metabolic diseases. Foods (Basel, Switzerland). (2021) 10:1854. doi: 10.3390/foods10081854 34441631 PMC8391153

[13] Kanarek N Petrova B Sabatini DM. Dietary modifications for enhanced cancer therapy. Nature. (2020) 579:507–17. doi: 10.1038/s41586-020-2124-0, PMID: 32214253

[14] Zhang J Lu X Wu R Ni H Xu L Wu W. Associations between composite dietary antioxidant index and estimated 10-year atherosclerotic cardiovascular disease risk among U.S. adults. Front Nutr. (2023) 10:1214875. doi: 10.3389/fnut.2023.1214875, PMID: 37637947 PMC10447978

[15] Chen X Lu H Chen Y Sang H Tang Y Zhao Y. Composite dietary antioxidant index was negatively associated with the prevalence of diabetes independent of cardiovascular diseases. Diabetol Metab Syndr. (2023) 15:183. doi: 10.1186/s13098-023-01150-6, PMID: 37684665 PMC10486118

[16] Zhao L Sun Y Cao R Wu X Huang T Peng W. Non-linear association between composite dietary antioxidant index and depression. Front Public Health. (2022) 10:988727. doi: 10.3389/fpubh.2022.988727, PMID: 36311643 PMC9609418

[17] Ma Y Liu J Sun J Cui Y Wu P Wei F. Composite dietary antioxidant index and the risk of heart failure: a cross-sectional study from NHANES. Clin Cardiol. (2023) 46:1538–43. doi: 10.1002/clc.24144, PMID: 37681461 PMC10716306

[18] Zhang J Chen Y Zou L Jin L Yang B Shu Y. Dose-response relationship between dietary antioxidant intake and diabetic kidney disease in the US adults with diabetes. Acta Diabetol. (2023) 60:1365–75. doi: 10.1007/s00592-023-02125-9, PMID: 37347448

[19] Borrud L Chiappa MM Burt VL Gahche J Zipf G Johnson CL. National Health and nutrition examination survey: national youth fitness survey plan, operations, and analysis. Vital and Health Statistics Series 2, Data Evaluation and Methods Res. (2012) 2014:1–24. 24709592

[20] Ahuja JK Moshfegh AJ Holden JM Harris E. USDA food and nutrient databases provide the infrastructure for food and nutrition research, policy, and practice. J Nutr. (2013) 143:241s–9s. doi: 10.3945/jn.112.170043, PMID: 23269654

[21] Jiang Y Shen Y. Composite dietary antioxidant index is inversely and nonlinearly associated with cardiovascular disease, atherosclerotic cardiovascular disease, and cardiovascular mortality in people with dyslipidemia: evidence from NHANES 2001-2018. Front Nutr. (2024) 11:1478825. doi: 10.3389/fnut.2024.1478825, PMID: 39845910 PMC11753228

[22] Wright ME Mayne ST Stolzenberg-Solomon RZ Li Z Pietinen P Taylor PR. Development of a comprehensive dietary antioxidant index and application to lung cancer risk in a cohort of male smokers. Am J Epidemiol. (2004) 160:68–76. doi: 10.1093/aje/kwh173, PMID: 15229119

[23] Maugeri A Hruskova J Jakubik J Kunzova S Sochor O Barchitta M. Dietary antioxidant intake decreases carotid intima media thickness in women but not in men: a cross-sectional assessment in the Kardiovize study. Free Radic Biol Med. (2019) 131:274–81. doi: 10.1016/j.freeradbiomed.2018.12.018, PMID: 30576781

[24] Nie K Deng T Bai Y Zhang Y Chen Z Peng X. Association between composite dietary antioxidant index and hyperlipidemia in adults based on the NHANES. Sci Rep. (2025) 15:2382. doi: 10.1038/s41598-025-86223-4, PMID: 39827264 PMC11742980

[25] Feng C Yao J Xie Y Yang F Fan X. Association between different composite dietary antioxidant indexes and low back pain in American women adults: a cross-sectional study from NHANES. BMC Public Health. (2024) 24:147. doi: 10.1186/s12889-024-17649-0, PMID: 38200420 PMC10782773

[26] Vasquez MM Hu C Roe DJ Chen Z Halonen M Guerra S. Least absolute shrinkage and selection operator type methods for the identification of serum biomarkers of overweight and obesity: simulation and application. BMC Med Res Methodol. (2016) 16:154. doi: 10.1186/s12874-016-0254-8, PMID: 27842498 PMC5109787

[27] Paul A Mukherjee DP Das P Gangopadhyay A Chintha AR Kundu S. Improved random Forest for classification. IEEE Transactions On Image Process: Pub IEEE Signal Processing Society. (2018) 27:4012–24. doi: 10.1109/TIP.2018.2834830, PMID: 29993742

[28] Koller D Pathak GA Wendt FR Tylee DS Levey DF Overstreet C. Epidemiologic and genetic associations of endometriosis with depression, anxiety, and eating disorders. JAMA Netw Open. (2023) 6:e2251214. doi: 10.1001/jamanetworkopen.2022.51214, PMID: 36652249 PMC9856929

[29] Smolarz B Szyłło K Romanowicz H. Endometriosis: epidemiology, classification, pathogenesis, treatment and genetics (review of literature). Int J Mol Sci. (2021) 22:10554. doi: 10.3390/ijms221910554, PMID: 34638893 PMC8508982

[30] Assaf L Eid AA Nassif J. Role of AMPK/mTOR, mitochondria, and ROS in the pathogenesis of endometriosis. Life Sci. (2022) 306:120805. doi: 10.1016/j.lfs.2022.120805, PMID: 35850246

[31] Falcone T Flyckt R. Clinical Management of Endometriosis. Obstet Gynecol. (2018) 131:557–71. doi: 10.1097/AOG.0000000000002469, PMID: 29420391

[32] Abramiuk M Mertowska P Frankowska K Świechowska-Starek P Satora M Polak G. How can selected dietary ingredients influence the development and progression of endometriosis? Nutrients. (2024) 16:154. doi: 10.3390/nu16010154, PMID: 38201982 PMC10781184

[33] Anderson G. Endometriosis Pathoetiology and pathophysiology: roles of vitamin a, estrogen, immunity, adipocytes, gut microbiome and Melatonergic pathway on mitochondria regulation. Biomol Concepts. (2019) 10:133–49. doi: 10.1515/bmc-2019-0017, PMID: 31339848

[34] Jiang Y Chen L Taylor RN Li C Zhou X. Physiological and pathological implications of retinoid action in the endometrium. J Endocrinol. (2018) 236:R169–r188. doi: 10.1530/JOE-17-0544, PMID: 29298821

[35] Li L Gao H Pan L Zhao Y Liang Z Zhang Q. All-trans retinoic acid inhibits epithelial-to-mesenchymal transition (EMT) through the down-regulation of IL-6 in endometriosis. Annals of Palliative Med. (2021) 10:11348–61. doi: 10.21037/apm-21-2175, PMID: 34872261

[36] Erten OU Ensari TA Dilbaz B Cakiroglu H Altinbas SK Çaydere M. Vitamin C is effective for the prevention and regression of endometriotic implants in an experimentally induced rat model of endometriosis. Taiwan J Obstet Gynecol. (2016) 55:251–7. doi: 10.1016/j.tjog.2015.07.004, PMID: 27125410

[37] Hoorsan H Simbar M Tehrani FR Fathi F Mosaffa N Riazi H. The effectiveness of antioxidant therapy (vitamin C) in an experimentally induced mouse model of ovarian endometriosis. Women's Health (Lond Engl). (2022) 18:17455057221096218. doi: 10.1177/17455057221096218, PMID: 35509242 PMC9087288

[38] Mier-Cabrera J Aburto-Soto T Burrola-Méndez S Jiménez-Zamudio L Tolentino MC Casanueva E. Women with endometriosis improved their peripheral antioxidant markers after the application of a high antioxidant diet. Reproductive Biol Endocrinol: RB&E. (2009) 7:54. doi: 10.1186/1477-7827-7-54, PMID: 19476631 PMC2693127

[39] Huang Y Wei Y Liang F Huang Y Huang J Luo X. Exploring the link between dietary zinc intake and endometriosis risk: insights from a cross-sectional analysis of American women. BMC Public Health. (2024) 24:2935. doi: 10.1186/s12889-024-20433-9, PMID: 39443887 PMC11515777

[40] Rabajdová M Špaková I Klepcová Z Smolko L Abrahamovská M Urdzík P. Zinc(II) niflumato complex effects on MMP activity and gene expression in human endometrial cell lines. Sci Rep. (2021) 11:19086. doi: 10.1038/s41598-021-98512-9, PMID: 34580366 PMC8476601

[41] Lu J Wang Z Cao J Chen Y Dong Y. A novel and compact review on the role of oxidative stress in female reproduction. Reproductive Biolog Endocrinol: RB&E. (2018) 16:80. doi: 10.1186/s12958-018-0391-5, PMID: 30126412 PMC6102891

[42] Amreen S Kumar P Gupta P Rao P. Evaluation of oxidative stress and severity of endometriosis. J Human Reproductive Sci. (2019) 12:40–6. doi: 10.4103/jhrs.JHRS_27_17, PMID: 31007466 PMC6472204

[43] Cirillo M Argento FR Becatti M Fiorillo C Coccia ME. Fatini C: Mediterranean diet and oxidative stress: a relationship with pain perception in endometriosis. Int J Mol Sci. (2023) 24:14601. doi: 10.3390/ijms241914601, PMID: 37834048 PMC10572576

[44] Oszajca K Adamus A. Diet in prevention and treatment of endometriosis: current state of knowledge. Current Nutrit Reports. (2024) 13:49–58. doi: 10.1007/s13668-024-00518-y, PMID: 38324218

[45] Amini L Chekini R Nateghi MR Haghani H Jamialahmadi T Sathyapalan T. The effect of combined vitamin C and vitamin E supplementation on oxidative stress markers in women with endometriosis: a randomized, triple-blind placebo-controlled clinical trial. Pain Res Manag. (2021) 2021:1–6. doi: 10.1155/2021/5529741, PMID: 34122682 PMC8172324

[46] Santanam N Kavtaradze N Murphy A Dominguez C Parthasarathy S. Antioxidant supplementation reduces endometriosis-related pelvic pain in humans. Translational Res: the J Laboratory Clin Med. (2013) 161:189–95. doi: 10.1016/j.trsl.2012.05.001, PMID: 22728166 PMC3484190

[47] Mier-Cabrera J Genera-García M De la Jara-Díaz J Perichart-Perera O Vadillo-Ortega F Hernández-Guerrero C. Effect of vitamins C and E supplementation on peripheral oxidative stress markers and pregnancy rate in women with endometriosis. Int J Gynaecol Obstet. (2008) 100:252–6. doi: 10.1016/j.ijgo.2007.08.018, PMID: 18005966

[48] Huijs E Nap A. The effects of nutrients on symptoms in women with endometriosis: a systematic review. Reprod Biomed Online. (2020) 41:317–28. doi: 10.1016/j.rbmo.2020.04.014, PMID: 32600946

[49] van Haaps AP Wijbers JV Schreurs AMF Vlek S Tuynman J De Bie B. The effect of dietary interventions on pain and quality of life in women diagnosed with endometriosis: a prospective study with control group. Hum Reprod. (2023) 38:2433–46. doi: 10.1093/humrep/dead214, PMID: 37877417 PMC10754387

[50] Hipólito-Reis M Neto AC Neves D. Impact of curcumin, quercetin, or resveratrol on the pathophysiology of endometriosis: a systematic review. Phytotherapy Res: PTR. (2022) 36:2416–33. doi: 10.1002/ptr.7464, PMID: 35583746

[51] Vallée A Lecarpentier Y. Curcumin and endometriosis. Int J Mol Sci. (2020) 21:2440.doi: 10.3390/ijms21072440, PMID: 32244563 PMC7177778

[52] Rostami S Alyasin A Saedi M Nekoonam S Khodarahmian M Moeini A. Astaxanthin ameliorates inflammation, oxidative stress, and reproductive outcomes in endometriosis patients undergoing assisted reproduction: a randomized, triple-blind placebo-controlled clinical trial. Front Endocrinol (Lausanne). (2023) 14:1144323. doi: 10.3389/fendo.2023.1144323, PMID: 37020589 PMC10067663

[53] Augoulea A Mastorakos G Lambrinoudaki I Christodoulakos G Creatsas G. The role of the oxidative-stress in the endometriosis-related infertility. Gynecol Endocrinol. (2009) 25:75–81. doi: 10.1080/09513590802485012, PMID: 19253102

[54] Kaltsas A Zikopoulos A Moustakli E Zachariou A Tsirka G Tsiampali C. The silent threat to Women's fertility: uncovering the devastating effects of oxidative stress. Antioxidants (Basel, Switzerland). (2023) 12:1490. doi: 10.3390/antiox12081490 37627485 PMC10451552

[55] Harlev A Gupta S Agarwal A. Targeting oxidative stress to treat endometriosis. Expert Opin Ther Targets. (2015) 19:1447–64. doi: 10.1517/14728222.2015.1077226, PMID: 26256952

[56] Saunders PTK Whitaker LHR Horne AW. Endometriosis: improvements and challenges in diagnosis and symptom management. Cell Reports Med. (2024) 5:101596. doi: 10.1016/j.xcrm.2024.101596, PMID: 38897171 PMC11228648

[57] Young SL. Nonsurgical approaches to the diagnosis and evaluation of endometriosis. Fertil Steril. (2024) 121:140–4. doi: 10.1016/j.fertnstert.2023.12.020, PMID: 38103884 PMC11149605

